# Supplementary material for: TGM6 is a helminth secretory product that mimics TGF-β binding to TGFBR2 to antagonize signaling in fibroblasts
Source: Nat Commun. 2025 Feb 21;16:1847. doi: 10.1038/s41467-025-56954-z (PMC11845725; doi:10.1038/s41467-025-56954-z)

Supplementary Information

TGM6, a helminth secretory product, mimics TGF-β binding to TGFBRII to antagonize signaling in fibroblasts

Stephen E. White, Tristin A. Schwartze, Ananya Mukundan, Christina Schoenherr, Shashi P. Singh, Maarten van Dinther, Kyle T. Cunningham, Madeleine P. J. White, Tiffany Campion, John Pritchard, Cynthia S. Hinck, Peter ten Dijke, Gareth J. Inman, Rick M. Maizels, and Andrew P. Hinck

10 Tables

12 Figures

Supplementary Table 1. TGM6:TGFBR2 binding as assessed by ITC[a]

Cell	32.3 μM TGM6	14.7 μM TGM6-D3
Syringe	339.7 μM TGFBR2	200.0 μM TGFBR2
Cell concentration (μM)	32.3	14.7
Temperature (°C)	25	35
N (sites)	1.16	1.01
K_D (nM) [a, c]	222 ± 97.1 [d]	437 ± 81 [e]
ΔH (kcal mol^{-1}) [b, c]	-8.918 ± 0.231 [d]	-21.268 ± 0.651 [e]
ΔG (kcal mol^{-1}) [c]	-9.066	-8.967
$-T\Delta S$ (kcal mol^{-1}) [c]	0.148	12.301

[a]Source data provided through Figshare [https://doi.org/10.6084/m9.figshare.28179359].
[b] Number of sites set to "1" for analysis.
[c] Uncertainty reported as ± 1σ.
[d]Global fit of two experiments.
[e]Global fit of three experiments.

Supplementary Table 2. Type II receptor binding to TGM6-D3 as assessed by ITC[a]

Cell	TGM6-D3	Buffer	TGM6-D3	Buffer	TGM6-D3	Buffer
Syringe	ActRII	ActRII	ActRIIb	ActRIIb	BMPRII	BMPRII
Cell concentration (μM)	7.35	0.00	10.0	0.00	20.0	0.00
Syringe concentration (μM)	60.5	60.5	100	100	215	215
Temperature (°C)	35	35	25	25	35	35
N (sites)	ND[b]	ND[b]	ND[b]	ND[b]	ND[b]	ND[b]
K_D (nM)	ND[b]	ND[b]	ND[b]	ND[b]	ND[b]	ND[b]
ΔH (kcal mol^{-1})	ND[b]	ND[b]	ND[b]	ND[b]	ND[b]	ND[b]
ΔG (kcal mol^{-1})	ND[b]	ND[b]	ND[b]	ND[b]	ND[b]	ND[b]
$-T\Delta S$ (kcal mol^{-1})	ND[b]	ND[b]	ND[b]	ND[b]	ND[b]	ND[b]

[a]Source data provided through Figshare [https://doi.org/10.6084/m9.figshare.28179359].
[b]Not determined due to weak or no signal

Supplementary Table 3. TGFBR2 competition binding as assessed by ITC[a]

Cell	15 μM TGF-β2-7M2R	15 μM TGM6-D3
Syringe	105 μM TGFBR2	150 μM TGFBR2
Competitor [b]	0.0 or 6.0 μM TGM6-D3	0.0 or 6.0 μM TGM1-D3
Temperature (°C)	35	25
K_D (nM)	19.34 (7.81, 39.57)[c]	188.6 (108.8, 308.0) [d]
ΔH (kcal mol^{-1})	-14.240 (-14.8531, -13.6366)[c]	-10.382 (-10.9570, -9.8751) [d]
ΔG (kcal mol^{-1})	-10.876[e]	-9.174[e]
-TΔS (kcal mol^{-1})	3.364[e]	1.208[f]

[a]Source data provided through Figshare [https://doi.org/10.6084/m9.figshare.28179359].
[b] Competitor was added to the sample cell.
[c]K_D and ΔH correspond to the parameters, derived from the global fit, for TGFBR2:mmTGF-β2-7M2R binding in the absence of competitor; uncertainty is reported as the limits of the ±1σ confidence interval.
[d] K_D and ΔH correspond to the parameters, derived from the global fit, for TGM6-D3:TGFBR2 binding in the absence of competitor; uncertainty is reported as the limits of the ±1σ confidence interval.
[e] ΔG and -TΔS correspond to those for TGFBR2:mmTGF-β2-7M binding in the absence of competitor calculated from ΔG = ΔH – TΔS and globally fitted values for K_D and ΔH.
[f] ΔG and -TΔS correspond to those for TGFBR2:TGM6-D3 binding in the absence of competitor calculated from ΔG = ΔH – TΔS and globally fitted values for K_D and ΔH.

Supplementary Table 4. TGM1-D45:mCD44 and TGM6-D45:mCD44 binding as assessed by ITC[a]

Syringe	Cell	Temp (°C)	N (sites)	K_D (nM)	ΔH (kcal mol^{-1})	ΔG (kcal mol^{-1})	-TΔS (kcal mol^{-1})
90 μM mCD44	8 μM TGM6-D45	35	ND	ND	ND	ND	ND
127 μM mCD44	15 μM TGM1-D45	35	0.82[b]	126 (76, 197)[c]	-32.4 (-34.4, -30.6)[c]	-9.7	22.7

[a]Source data provided through Figshare [https://doi.org/10.6084/m9.figshare.28179359].
[b]Number of sites determined by incompetent fraction value on Sedphat; set to '1' for thermodynamic analysis.
[c]Uncertainty reported as 68.3% confidence interval.

Supplementary Table 5. Crystallographic data, phasing, and refinement of the TGM6-D3:TGFBR2 complex[a]

**Data Collection**	
X-ray Source	APS BEAMLINE 22-ID
Wavelength	1.00 Å
Detector	DECTRIS EIGER X 16M
**Data Reduction[a]**	
Space Group	$P2_12_12$
a,b,c (Å)	56.04, 128.39, 29.65
α,β,γ (°)	90, 90, 90
Completeness Overall (%)	98.5 (85.1)
Resolution (Å)	42.80-1.40 (7.67-1.40)
R_{meas}	0.079 (1.428)
R_{pim}	0.022 (0.506)
$\langle I/\sigma(I) \rangle$	17.5 (1.6)
Redundancy or Multiplicity	12.4 (7.5)
$CC_{1/2}$	0.999 (0.700)
CC*	1.000 (0.907)
Matthews Coefficient	2.43
No. Observations	528172
No. Reflections	42468
**Refinement**	
1:1 complexes in the asymmetric unit	1
Resolution (Å)	42.253 - 1.401
R_{work} / R_{free}	0.1596 / 0.1824
Residues	110, 83
Atoms / Non-Hydrogens	3462 / 1896
Protein Overall / Heavy / Backbone	3074 / 1585 / 780
Ion and Ligand Overall / Heavy	153 / 76
Water(s)	235
B-factors	37.17
Protein Overall / Heavy / Backbone	36.98 / 32.98 / 28.73
Ion and Ligand Overall / Heavy	33.78 / 32.29
Water(s)	41.81
R.M.S. Deviations	
Bond-Lengths	0.0100
Bond-Angles	1.5016
MolProbity	
Clash Score	3.44
MolProbity Score	1.41
Ramachandran	
Allowed	98.93 %
Favored	96.26 %
Outliers	1.07 %
Rotamers	
Allowed	100%
Favored	98.92 %
Outliers	1.08 %
Clashes	1
**Deposition**	
RCSB PDB	9E9G [https://doi.org/10.2210/pdb9E9G/pdb]

[a]The numbers in the Data Reduction section correspond to the value for the overall and in parentheses the high resolution shell

Supplementary Table 6. TGM6-D3 WT:TGFBR2 variant and TGFBR2 WT:TGM6-D3 variant binding as assessed by ITC at 25 °C[a]

Syringe	Cell	Temp (°C)	N (sites)[b]	K_D (μM)[c]	ΔH (kcal mol^{-1})[c]	ΔG (kcal mol^{-1})	-TΔS (kcal mol^{-1})
426 μM TGM6-D3	25 μM TGFBR2 WT	25	0.68	0.35 (0.28, 0.43)	-16.3 (-16.8, -15.9)	-8.8	7.5
590 μM TGM6-D3	30 μM TGFBR2 D55A	35	0.52	2.25 (2.05, 2.45)	-22.1 (-22.6, -21.5)	-8.0	14.1
290 μM[a] TGM6-D3	18.4 μM TGFBR2 I76A	25	0.69	7.35 (6.40, 8.51)	-10.6 (-11.4, -10.0)	-7.0	3.6
290 μM[a] TGM6-D3	18.9μM TGFBR2 D141A	25	0.75	0.94 (0.87 1.00)	-11.5 (-11.7, -11.4)	-8.2	3.3
590 μM[a] TGM6-D3	30 μM TGFBR2 E142A	35	0.56	3.17 (2.75, 3.67)	-21.8 (-23.1, -20.8)	-7.8	14.1
300 μM TGFBR2	10 μM TGM6-D3 WT	25	0.87	0.36 (0.31, 0.41)	-11.3 (-11.6, -11.1)	-8.8	2.5
667 μM TGFBR2	25 μM TGM6-D3 R38A	25	0.85	8.03 (7.61, 8.48)	-17.0 (-17.4, -16.6)	-7.0	10.0
667 μM TGFBR2	25 μM TGM6-D3 I78A	25	0.71	5.56 (5.24, 5.71)	-17.5 (-17.6, -17.3)	-7.2	10.3
667 μM TGFBR2	25 μM TGM6-D3 Y80A	25	0.33	48.4 (38.1, 61.8)	-21.5 (-26.6, -17.7)	-5.9	15.6
150 μM TGFBR2	10 μM TGM6-D3 Y80F	25	0.67	1.25 (1.04, 1.49)	-9.6 (-10.2, -9.1)	-8.1	1.6
200 μM TGFBR2	10 μM TGM6-D3 R82A	35	0.93	4.23 (3.45, 5.27)	-16.6 (-18.9, -15.0)	-7.6	9.1
200 μM TGFBR2	10 μM TGM6-D3 R82S	35	1.12	3.09 (2.37, 4.11)	-16.6 (-18.8, -15.1)	-7.9	8.8
667 μM TGFBR2	25 μM TGM6-D3 Y93A	25	0.17	39.0 (30.4, 50.8)	-19.2 (-23.2, -16.3)	-6.0	13.2
150 μM TGFBR2	10 μM TGM6-D3 R95A	25	1.00	3.82 (1.50, 15.18)	-13.3 (-30.3, -9.4)	-7.4	5.9
150 μM TGFBR2	10 μM TGM6-D3 P94K R95N (KN)	25	0.90	0.27 (0.23, 0.30)	-13.4 (-13.6, -13.1)	-9.0	4.4
120 μM TGFBR2	10 μM TGM6-D3 Q81K R82S R83G G84T (KSGT)	35	0.82	4.49 (1.74, 9.76)[d]	-21.6 (-39.6, -14.2)[d]	-7.5	14.0
100 μM TGFBR2	10 μM TGM1-D3	35	0.72	1.53 (0.68, 4.48)	-9.3 (-15.2, -7.2)	-8.2	1.1
360 μM TGFBR2	30 μM TGM1-D3 S242R	35	0.70	0.38 (0.24, 0.56)	-11.6 (-12.4, -10.9)	-9.1	2.5
120 μM TGFBR2	10 μM TGM1-D3 K254P N255R (PR)	35	0.91	2.62 (0.39, 28.11)[e]	-5.2 (-9.3, -3.4)[e]	-7.9	-2.6
120 μM TGFBR2	10 μM TGM1-D3 K241Q S242R G243R T244G (QRRG)	35	1.10	0.17 (0.07, 0.40)	-8.4 (-9.5, -7.4)	-9.5	-1.13

[a]Source data provided through Figshare [https://doi.org/10.6084/m9.figshare.28179359].
[b] Number of sites determined by incompetent fraction value on Sedphat; set to '1' for thermodynamic analysis.
[c] Uncertainty reported as 68.3% confidence interval.
[d] Fit by constraining either the ΔH to -31.5, -11.5 or the K_D^{-1} to (5.05, 5.65) x 10^5
[e] Fit by constraining either the ΔH to -15.2, -0.2 or the K_D^{-1} to (5.28, 5.88) x 10^5

Supplementary Table 7. *H. polygyrus* TGM constructs used in this study

Construct	Residue range and features*	Sequence
TGM6	Residues 17-254 of *H. polygyrus* TGF-β Mimic 6 (NCBI MG429741)<br><br>Expressed as Igk Signal Peptide-TGM6-Linker-Myc Tag-Linker-His6 fusion	METDTLLLWV LLLWVPGSTG DAAQPARRAS CPPLPDDETV WYEYYGYVDG RHTVGDAAIK DSLENYPPNT HARRHCKALS KKADPGEFVA ICYQRRGTSE SQWQYYPRIA SCPDPRCKPL EKNDSVSYEY FTKPTKGLKM GSITKPDKSG KYPEETFVRR YCNDLPRNSL AQGKTYAECL DSEWKLKNLP DCRFAAGCDE EYLLEKLMFV DISYWGKDAA KFSDDKTYRY YRPGSKVTAK CKGKSVKLTC VDGGYWVTVD GRKALCTAAA RGGPEQKLIS EEDLNSAVDH HHHHH
TGM6-D3	Residues 15-102 of *H. polygyrus* TGF-β Mimic 6 (NCBI MG429741)<br><br>Expressed as a Thioredoxin- His10-Linker-Thrombin-Linker-TGM6-D3 fusion	MSDKIIHLTD DSFDTDVLKA DGAILVDFWA EWCGPCKMIA PILDEIADEY QGKLTVAKLN IDQNPGTAPK YGIRGIPTLL LFKNGEVAAT KVGALSKGQL KEFLDANLAG SGSGHMSSGH HHHHHHHHHS SGGSGLVPR|G SGTGSSCPPL PDDETVWYEY YGYVDGRHTV GDAAIKDSLE NYPPNTHARR HCKALSKKAD PGEFVAICYQ RRGTSESQWQ YYPRIASCPD P
TGM6-D45	Residues 103-254 of *H. polygyrus* TGF-β Mimic 6 (NCBI MG429741)<br><br>Expressed as a Thioredoxin- His10-Linker-Thrombin-Linker-TGM-D45 fusion	MSDKIIHLTD DSFDTDVLKA DGAILVDFWA EWCGPCKMIA PILDEIADEY QGKLTVAKLN IDQNPGTAPK YGIRGIPTLL LFKNGEVAAT KVGALSKGQL KEFLDANLAG SGSGHMSSGH HHHHHHHHHS SGGSGLVPR|G SGTRCKPLEK NDSVSYEYFT KPTKGLKMGS ITKPDKSGKY PEETFVRRYC NDLPRNSLAQ GKTYAECLDS EWKLKNLPDC RFAAGCDEEY LLEKLMFVDI SYWGKDAAKF SDDKTYRYYR PGSKVTAKCK GKSVKLTCVD GGYWVTVDGR KALCT
TGM1	Residues 16-422 of *H. Polygyrus* TGF-β Mimic 1 (NCBI ATO59092.1)<br><br>Expressed as Igk Signal Peptide-Linker-TGM1-Linker-Myc Tag-Linker-His6 fusion	METDTLLLWVLLLWVPGSTGDAAQPARRADDSGCMPFSDEAAT YKYVAKGPKNIEIPAQIDNSGMYPDYTHVKRFCKGLHGEDTTG WFVGICLASQWYYYEGVQECDDRRCSPLPTNDTVSFEYLKATV NPGIIFNITVHPDASGKYPELTYIKRICKNFPTDSNVQGHIIG MCYNAEWQFSSTPTCPASGCPPLPDDGIVFYEYYGYAGDRHTV GPVVTKDSSGNYPSPTHARRRCRALSQEADPGEFVAICYKSGT TGESHWEYYKNIGKCPDPRCKPLEANESVHYEYFTMTNETDKK KGPPAKVGKSGKYPEHTCVKKVCSKWPYTCSTGGPIFGECIGA TWNFTALMECINARGCSSDDLFDKLGFEKVIVRKGEGSDSYKD DFARFYATGSKVIAECGGKTVRLECSNGEWHEPGTKTVHRCTK DGIRTLGPEQKLISEEDLNSAVDHHHHHH-
TGM1-D123	Residues 16-262 of *H. Polygyrus* TGF-β Mimic 1 (NCBI ATO59092.1)<br>Expressed as Igk Signal Peptide-Linker-TGM1-D123-Linker-Myc Tag-Linker-His6 fusion	METDTLLLWVLLLWVPGSTGDAAQPARRADDSGCMPFSDEAAT YKYVAKGPKNIEIPAQIDNSGMYPDYTHVKRFCKGLHGEDTTG WFVGICLASQWYYYEGVQECDDRRCSPLPTNDTVSFEYLKATV NPGIIFNITVHPDASGKYPELTYIKRICKNFPTDSNVQGHIIG MCYNAEWQFSSTPTCPASGCPPLPDDGIVFYEYYGYAGDRHTV GPVVTKDSSGNYPSPTHARRRCRALSQEADPGEFVAICYKSGT TGESHWEYYKNIGKCPDPGPEQKLISEEDLNSAVDHHHHHH-

TGM1-D3	Residues 177-262 of *H. Polygyrus* TGF-β Mimic 1 (NCBI ATO59092.1)<br><br>Expressed as a Thioredoxin- His6-Linker-Thrombin-Linker-TGM-D3 fusion	MSDKIIHLTD DSFDTDVLKA DGAILVDFWA EWCGPCKMIA PILDEIADEY QGKLTVAKLN IDQNPGTAPK YGIRGIPTLL LFKNGEVAAT KVGALSKGQL KEFLDANLAG SGSGHMHHHH HHSSGLVPR|G SGTGCPPLPD DGIVFYEYYG YAGDRHTVGP VVTKDSSGNY PSPTHARRRC RALSQEADPG EFVAICYKSG TTGESHWEYY KNIGKCPDP

*All residue numbering begins with the N-terminal methionine of the naturally occurring signal peptide

Supplementary Table 8. Type I receptor constructs used in this study

Construct	Residue range and features*	Sequence
ALK1 (TSRI)	Residues 22-118 of human ALK1 (TSR1) (NCBI NP_000011)<br><br>Expressed as a Linker-His6-Linker-Thrombin-Linker-Alk1 fusion	MGSSHHHHHH SSGLVPR|GSH MDPVKPSRGP LVTCTCESPH CKGPTCRGAW CTVVLVREEG RHPQEHRGCG NLHRELCRGR PTEFVNHYCC DSHLCNHNVS LVLEATQPPS EQPGTDGQ
ALK2 (ActRIA)	Residues 21-120 of human ALK2 (ActRIA) (NCBI NP_001096)<br><br>Expressed as a Thioredoxin-His6-Linker-Thrombin-Linker-Alk2 fusion	MSDKIIHLTD DSFDTDVLKA DGAILVDFWA EWCGPCKMIA PILDEIADEY QGKLTVAKLN IDQNPGTAPK YGIRGIPTLL LFKNGEVAAT KVGALSKGQL KEFLDANLAG SGSGHMHHHH HHSSGLVPR|G SGTMEDEKPK VNPKLYMCVC EGLSCGNEDH CEGQQCFSSL SINDGFHVYQ KGCFQVYEQG KMTCKTPPSP GQAVECCQGD WCNRNITAQL PTKGKSFPGT QNF
ALK3 (BMPRIA)	Residues 24-152 of human ALK3 (BMPRIA) (NCBI NP_001393488)<br><br>Expressed as a Thioredoxin-His6-Linker-Thrombin-Linker-Alk3 fusion	MSDKIIHLTD DSFDTDVLKA DGAILVDFWA EWCGPCKMIA PILDEIADEY QGKLTVAKLN IDQNPGTAPK YGIRGIPTLL LFKNGEVAAT KVGALSKGQL KEFLDANLAG SGSGHMHHHH HHSSGLVPR|G SGTQNLDSML HGTGMKSDSD QKKSENGVTL APEDTLPFLK CYCSGHCPDD AINNTCITNG HCFAIIEEDD QGETTLASGC MKYEGSDFQC KDSPKAQLRR TIECCRTNLC NQYLQPTLPP VVIGPFFDGS IR
ALK4 (ActRIB)	Residues 29-107 of human ALK4 (ActRIB) (NCBI NP_001399711)<br><br>Expressed as a Linker-His6-Linker-Thrombin-Linker-Alk4 fusion	MGSSHHHHHH SSGLVPR|GSH MVQALLCACT SCLQANYTCE TDGACMVSIF NLDGMEHHVR TCIPKVELVP AGKPFYCLSS EDLRNTHCCY TDYCNRIDLR
ALK5 (TGFBR1)	Residues 25-125 of human ALK5 (TGFBRI) (NCBI NP_004603.1)<br><br>Expressed as Linker-His6-Linker-Thrombin-Linker-TbRI fusion	MGSSHHHHHH SSGLVPR|GSH MAALLPGATA LQCFCHLCTK DNFTCVTDGL CFVSVTETTD KVIHNSSCIA EIDLIPRDRP FVCAPSSKTG SVTTTYCCNQ DHCNKIELPT TVKSSPGLGP VE

*All residue numbering begins with the N-terminal methionine of the naturally occurring signal peptide

Supplementary Table 9. Type II receptor constructs used in this study

Construct	Residue range and features*	Sequence
ActRII	Residues 20-121 of the human Activin type II receptor (NCBI NP_001265508)<br><br>Expressed as Linker-His6-Linker-Thrombin-Linker-ActRII fusion	MGSSHHHHHH SSGLVPR|GSH MAILGRSETQ ECLFFNANWE KDRTNQTGVE PCYGDKDKRR HCFATWKNIS GSIEIVKQGC WLDDINCYDR TDCVEKKDSP EVYFCCCEGN MCNEKFSYFP EME
ActRIIb	Residues 25-117 of the human Activin type IIb receptor (NCBI NP_001097)<br><br>Expressed as Linker-His6-Linker-Thrombin-Artifact-Linker-ActRIIb fusion	MGSSHHHHHH SSGLVPR|GSH MLEDPVPETR ECIYYNANWE LERTNQSGLE RCEGEQDKRL HCYASWRNSS GTIELVKKGC WLDDFNCYDR QECVATEENP QVYFCCCEGN FCNERFTHLP
BMPRII	Residues 29-133 of the human BMP type II receptor (NCBI NP_001195)<br><br>Expressed as Signal-Linker-His6-Linker-Thrombin-Linker-BMPRII fusion	MKWVTFLLLL FISGSAFSAA AGSSHHHHHH SSGLVPR|GSH MNQERLCAFK DPYQQDLGIG ESRISHENGT ILCSKGSTCY GLWEKSKGDI NLVKQGCWSH IGDPQECHYE ECVVTTTPPS IQNGTYRFCC CSTDLCNVNF TENFPP
TGFBR2	Residues 38-153 of the human TGF-β type II receptor (NCBI NP_003233)<br><br>Expressed as TGFBRII alone, with no tags or otherwise	MVTDNNGAVK FPQLCKFCDV RFSTCDQKSC MSNCSITSIC EKPQEVCVAV WRKNENITLE TVCHDPKLPY HDFILEDAAS PKCIMKEKKK PGETFFMCSC SSDECNDNII FSEEY

*All residue numbering begins with the N-terminal methionine of the naturally occurring signal peptide

Supplementary Table 10. Growth factor constructs used in this study

Construct	Residue range and features*	Sequence
mmTGF-β2-7M2R	Residues 303-352 and 377-414 of mouse TGF-β2 (NCBI NP_0033393) connected by an engineered loop<br><br>C379R substitution renders the protein monomeric; K327R, R328K, V381R, L391V, I394V, K396R, T397K, and I400V substitutions enable high affinity TGFBR2 binding and high solubility	ALDAAYCFRN VQDNCCLRPL YIDFRKDLGW KWIHEPKGYN ANFCAGACPY RASKSPRCRS QDLEPLTIVY YVGRKPKVEQ LSNMIVKSCK CS
mCD44	Residues 23 -174 of mouse CD44 (NCBI XP_006498709)<br><br>Expressed as Signal-His6-Linker-Thrombin-mCD44	MKWVTFLLLL FISGSAFSGS HHHHHHGSLV PRGSHQQIDL NVTCRYAGVF HVEKNGRYSI SRTEAADLCQ AFNSTLPTMD QMKLALSKGF ETCRYGFIEG NVVIPRIHPN AICAANHTGV YILVTSNTSH YDTYCFNASA PPEEDCTSVT DLPNSFDGPV TITIVNRDGT RYSKKGEYRT HQEDID

*All residue numbering begins with the N-terminal methionine of the naturally occurring signal peptide

Supplementary Figure 1. TGM6-D45 is natively folded. The ^{1}H-^{15}N HSQC spectrum of ^{15}N-labeled TGM6-D45 has many signals outside of the random coil region (7.8 – 8.6 ppm ^{1}H), demonstrating that it is natively folded. Source data are provided through Figshare [https://doi.org/10.6084/m9.figshare.28179359].

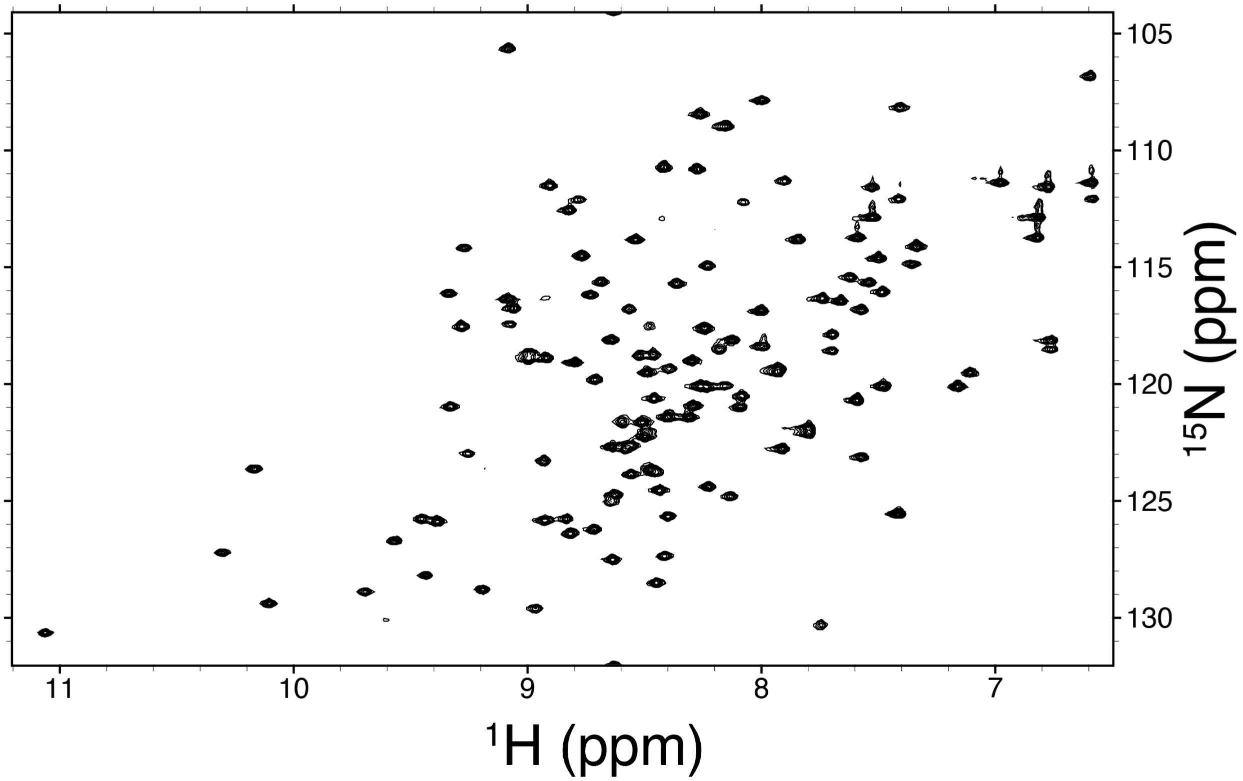

Supplementary Figure 2. TGM6-D3 does not bind ActRII, ActRIIb, or BMPRII. a-f Thermograms obtained upon the injection of ActRII, ActRIIb, or BMPRII into TGM6-D3 or Buffer. Panels (**a, c, e**) correspond to the injection of ActRII, ActRIIb, and BMPRII into TGM6-D3, respectively; panels (**b, d, f**) correspond to the injection of ActRII, ActRIIb, and BMPRII into buffer, respectively. Source data of (**a-f**) are provided through Figshare [https://doi.org/10.6084/m9.figshare.28179359].

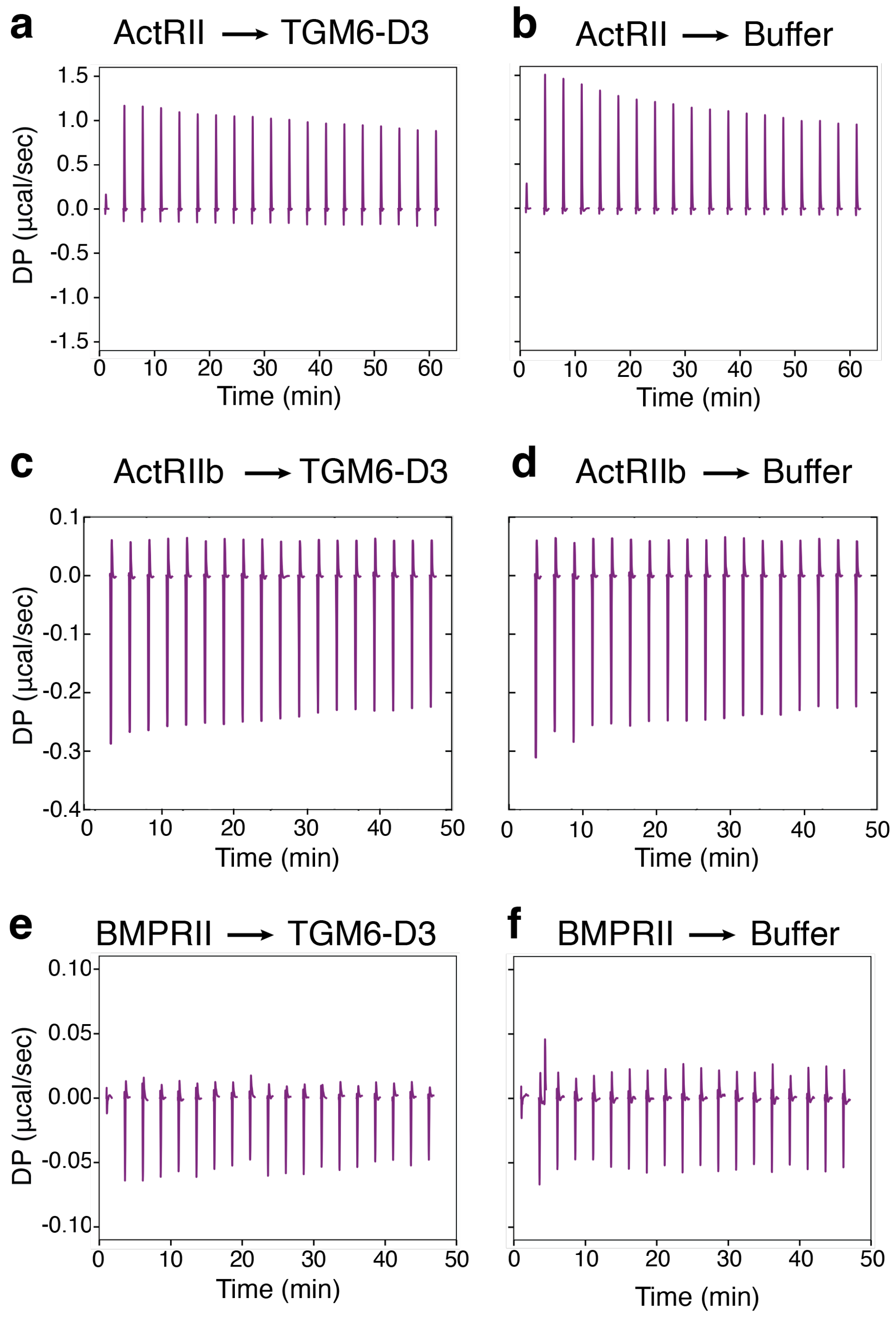

Supplementary Figure 3. TGM6-D45 does not bind to any type I receptors. a-d ^{1}H-^{15}N HSQC spectra of ^{15}N-labeled type I receptors bound to an excess of unlabeled TGM6-D45 (red) overlaid onto the spectra of the type I receptors alone (blue). The receptors tested were: (**a**) ^{15}N-ALK1; (**b**) ^{15}N-ALK2; (**c**) ^{15}N-ALK3; and (**d**) ^{15}N-ALK4. Source data of (**a-d**) are provided through Figshare [https://doi.org/10.6084/m9.figshare.28179359].

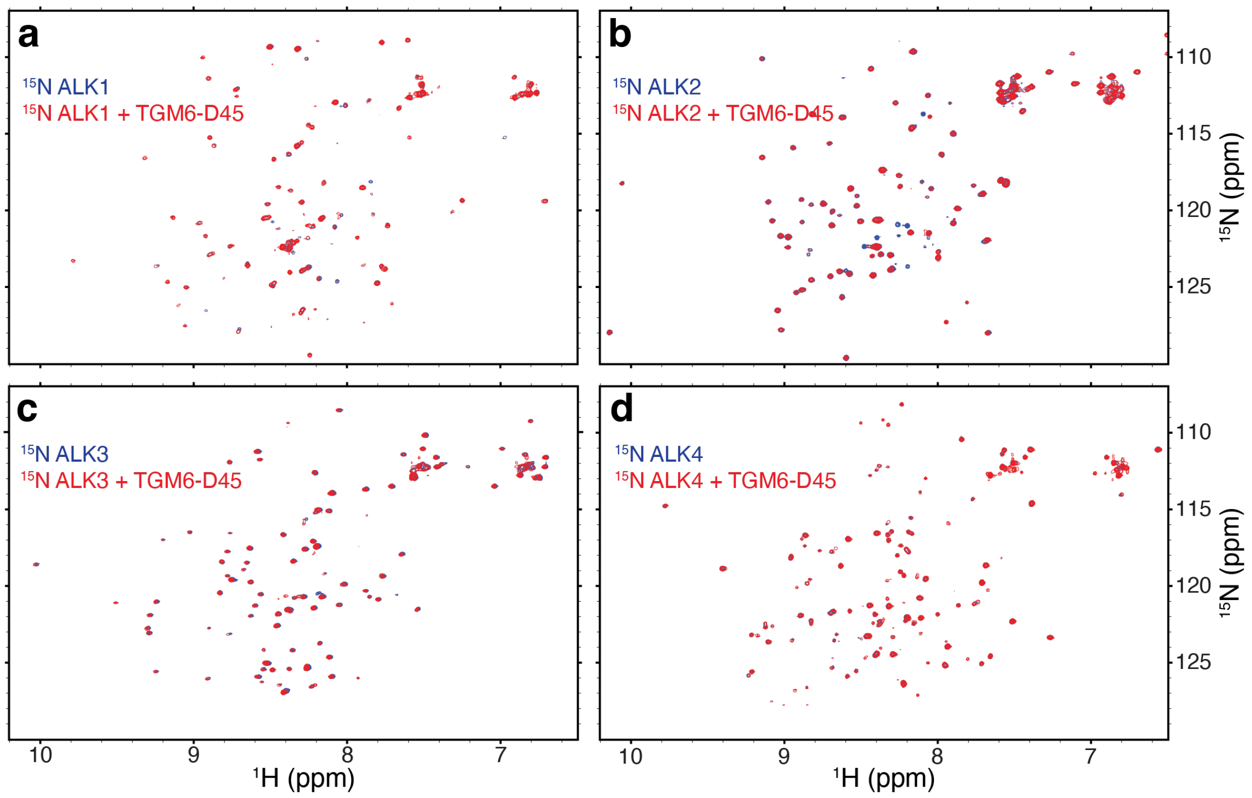

Supplementary Figure 4. The TGM6:TGFBR2 binary complex does not bind to any type I receptors. **a-d** ^{1}H-^{15}N HSQC spectra of ^{15}N-labeled type I receptors as bound to an excess of unlabeled TGM6:TGFBR2 binary complex (red) overlaid onto the spectra of the type I receptors alone (blue). The receptors tested were: (**a**) ^{15}N-ALK1; (**b**) ^{15}N-ALK2; (**c**) ^{15}N-ALK3; and (**d**) ^{15}N-ALK4. Source data of (**a-d**) are provided through Figshare [https://doi.org/10.6084/m9.figshare.28179359].

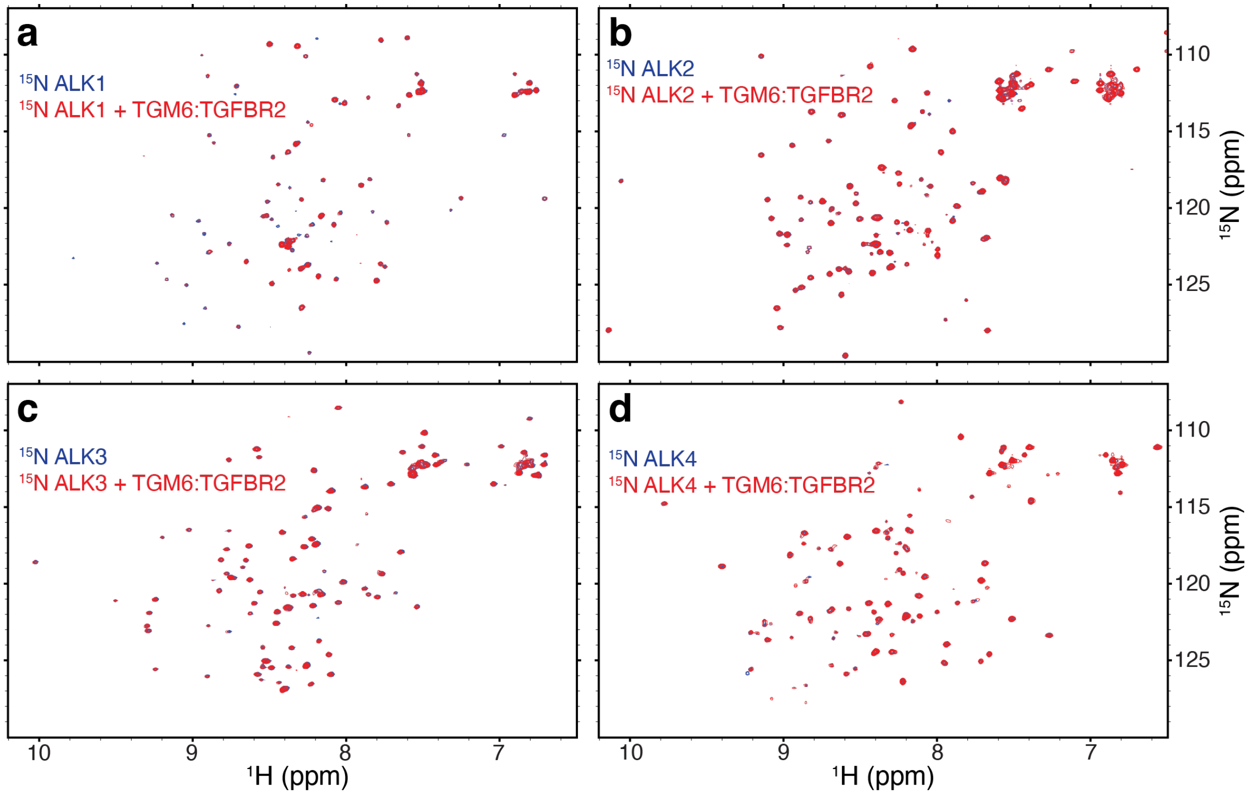

Supplementary Figure 5. Mass spectra and 1D ^{1}H NMR spectra confirming the identity and native folding of ActRII, ActRIIb, and BMPRII used in the ITC experiments. a-f 1D ^{1}H NMR spectrum and intact mass spectrum of ActRII ((**a**) and (**b**), respectively), ActRIIb ((**c**) and (**d**), respectively), and BMPRII ((**e**) and (**f**), respectively). Source data of (**a-f**) are provided through Figshare [https://doi.org/10.6084/m9.figshare.28179359].

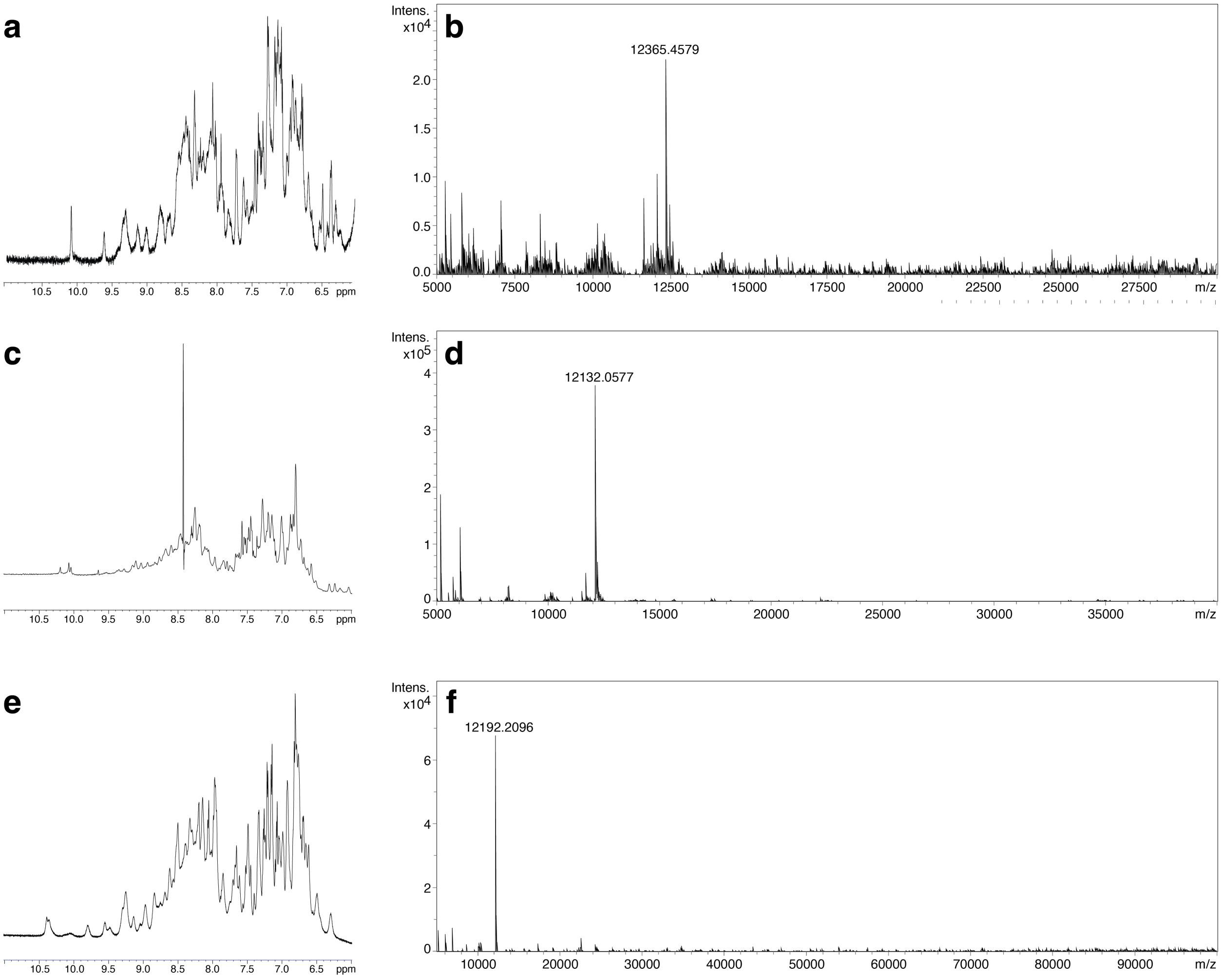

Supplementary Figure 6. Inhibition of TGF-β1 signaling in mouse EL4 T-cells and mouse NM18 breast epithelial cells. a-b Inhibition of TGF-β1 signaling in mouse MFB-F11 fibroblasts (**a**) or mouse EL4-T cells (**b**) by TGM6 as detected by pSMAD2 Western blotting. **c** Inhibition of TGF-β3 signaling in mouse NM18 breast epithelial cells or mouse NIH3T3 fibroblasts containing the CAGA-dynGFP reporter (Marvin, et. al (2022) Cancers, 14, 2508). Cells were seeded in 96-well plates and pre-treated for 30 minutes with different doses of TGM6 (0.04, 0.4, and 1 nM). Cells were subsequently stimulated with 40 pM TGF-β3. The cells were subsequently imaged every 3 hours for a period of 48 hours in an IncuCyte S3 live-cell imaging analysis system (Sartorius). Relative reporter activity at peak stimulation (18h) is shown. The depicted mean response was derived from one experiment with quadripulate measurements for each treatment condition and are normalized to that of cells treated with TGF-β3 alone. Source data of (**c**) provided as a Source Data file. Uncropped blots (**a, b**) provided at the end of the Supplementary Information file.

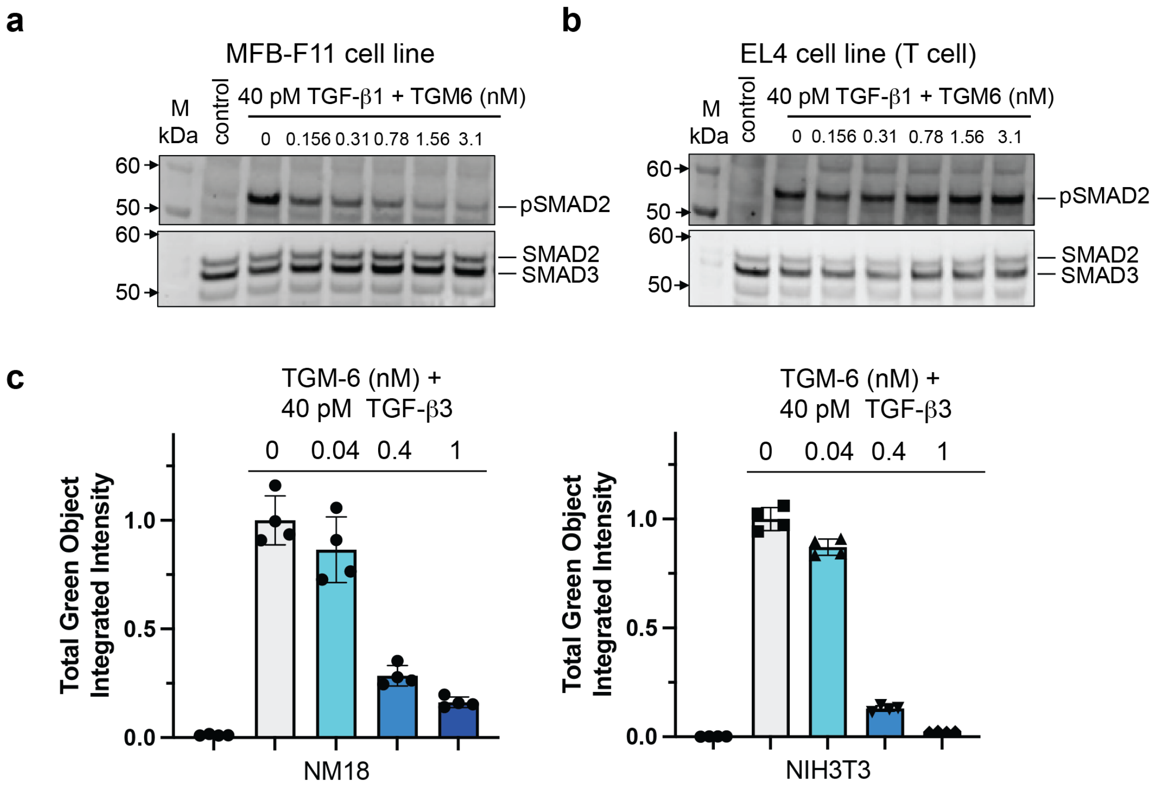

Supplementary Figure 7. TGM6 does not stimulate BMP activity nor does it inhibit BMP or activin signaling activity. a Stimulation of BMP mCherry reporter in NIH3T3 cells by BMP2, BMP6, and BMP7, and TGM6 at the concentrations shown in either the absence or presence of 3.6 nM TGM-6. **b** Stimulation of TGF-β/Activin GFP reporter in NIH3T3 cells by activinA or GDF8 at the concentrations shown in either the absence or presence of 3.6 nM TGM-6. Data shown in (**a**) and (**b**) are mean and standard deviation of 3 – 5 replicate measurements from three (BMP) and three (ActA) experiments with similar results. Source data of (**a-b**) provided as a Source Data file.

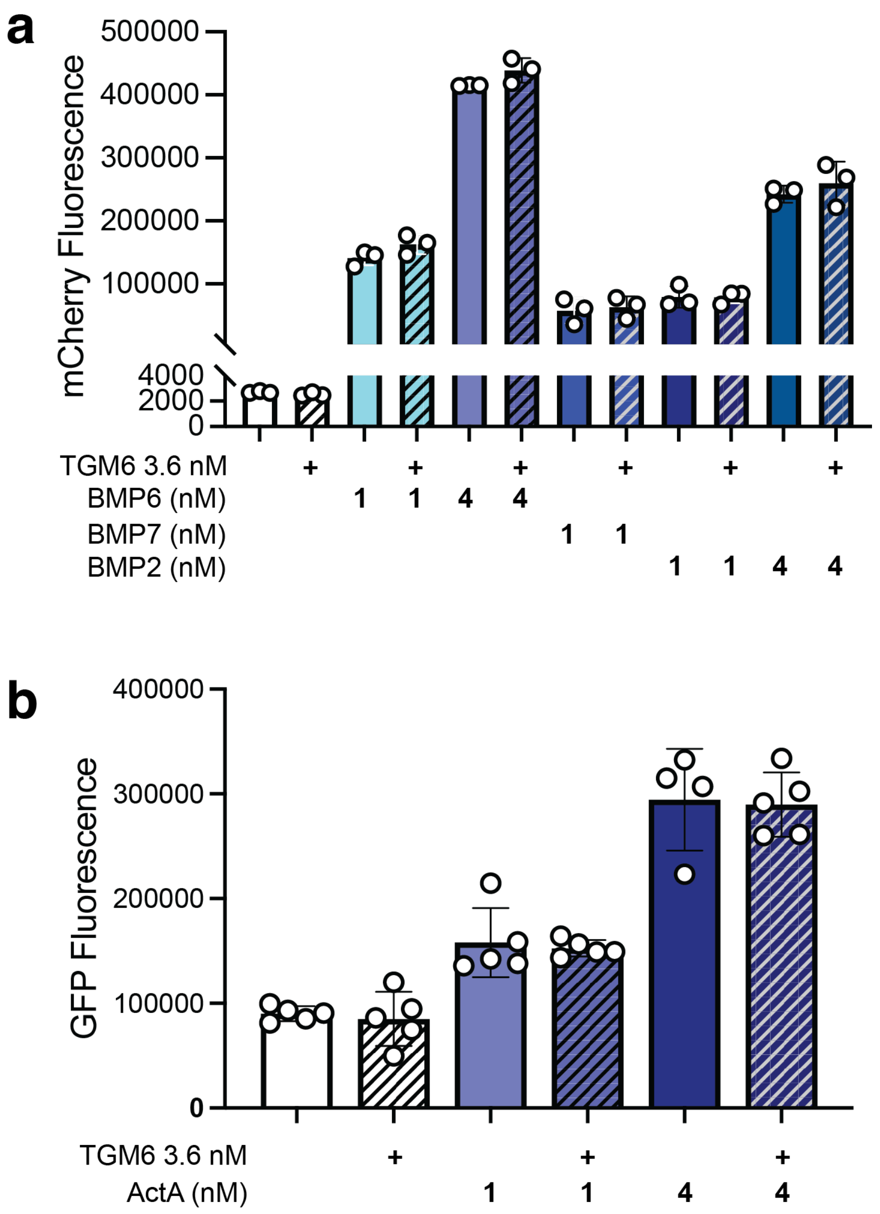

Supplementary Figure 8. Overlay of the TGFBR2 and TGM6-D3 components of the TGFBR2:TGM6-D3 crystal structure with their closest unbound counterpart. a Overlay of the TGFBR2 component of the TGFBR2:TGM6-D3 crystal structure with the crystal structure of unbound TGFBR2 (PDB 1M9Z). **b** Overlay of the TGM6-D3 component of the TGFBR2:TGM6-D3 crystal structure with the lowest energy member of the ensemble of TGM1-D3 solution structures (PDB 7SXB). Structural data is available through the RCSB PDB under accession code 9E9G [https://doi.org/10.2210/pdb9E9G/pdb].

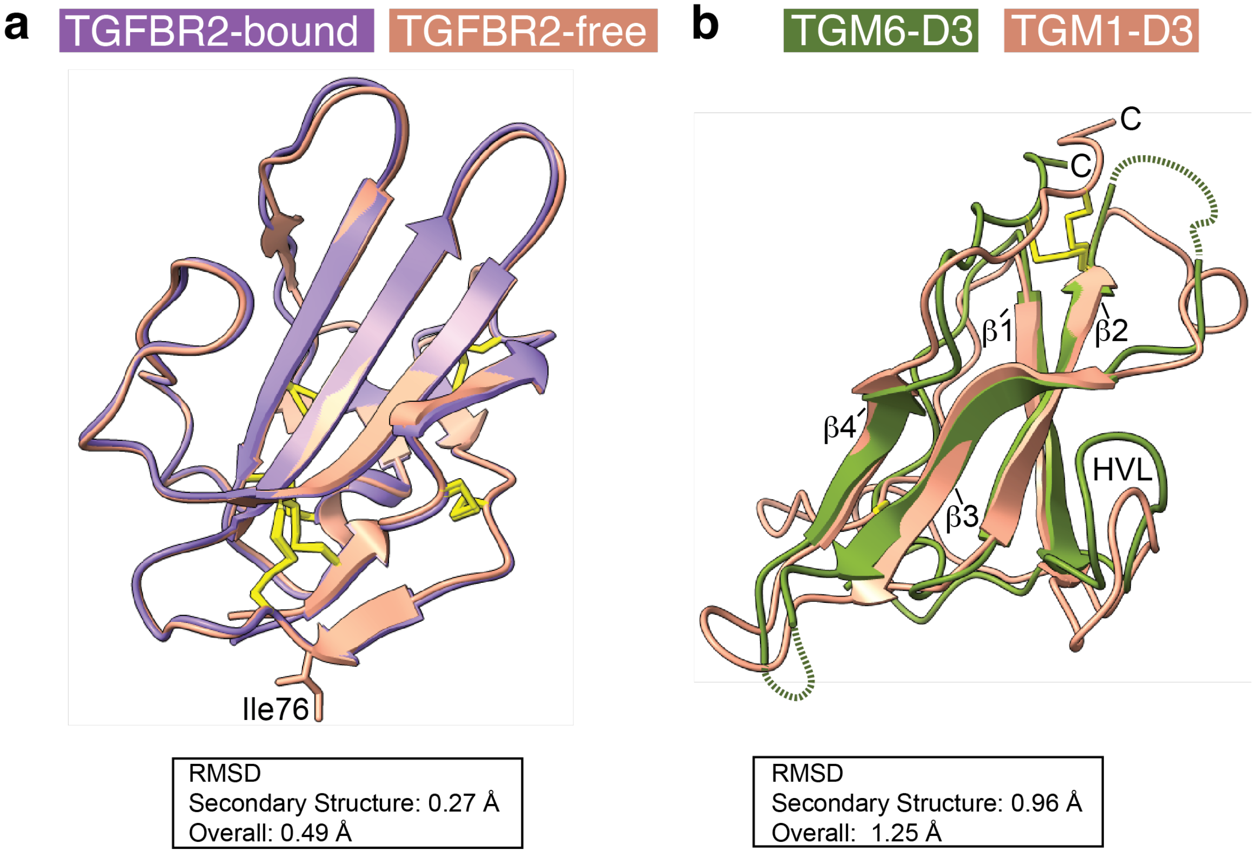

Supplementary Figure 9. ITC thermograms obtained for WT TGM6-D3 injected into TGFBR2 variants (A-E) and WT TGFBR2 injected into TGM6-D3 variants (F-P) or TGM1-D3 variants (Q-T). a TGFBR2 WT, **b** TGFBR2 D55A, **c** TGFBR2 I76A, **d** TGFBR2 D141A, **e** TGFBR2 E142A, **f** TGM6-D3 WT, **g** TGM6-D3 R38A, **h** TGM6-D3 I78A, **i** TGM6-D3 Y80A, **j** TGM6-D3 Y80F, **k** TGM6-D3 R82A, **l** TGM6-D3 R82S, **m** TGM6-D3 Y93A, **n** TGM6-D3 R95A, **o** TGM6-D3 P94K R95N (KN), **p** TGM6-D3 Q81K R82S R83G G84T (KSGT), **q** TGM1-D3 WT, **r** TGM1-D3 S242R, **s** TGM1-D3 K254P N255R (PR), and t TGM1-D3 K241Q S242R G243R T244G (QRRG). The binding experiments were fit to a 1:1 binding model from one (**a, c, d, f, g, h, i, j, m, n, o**) or two (**b, e, k, l, p, q, r, s, t**) experiments (purple and purple and blue, respectively) per variant. Source data of (**a-t**) are provided through Figshare [https://doi.org/10.6084/m9.figshare.28179359].

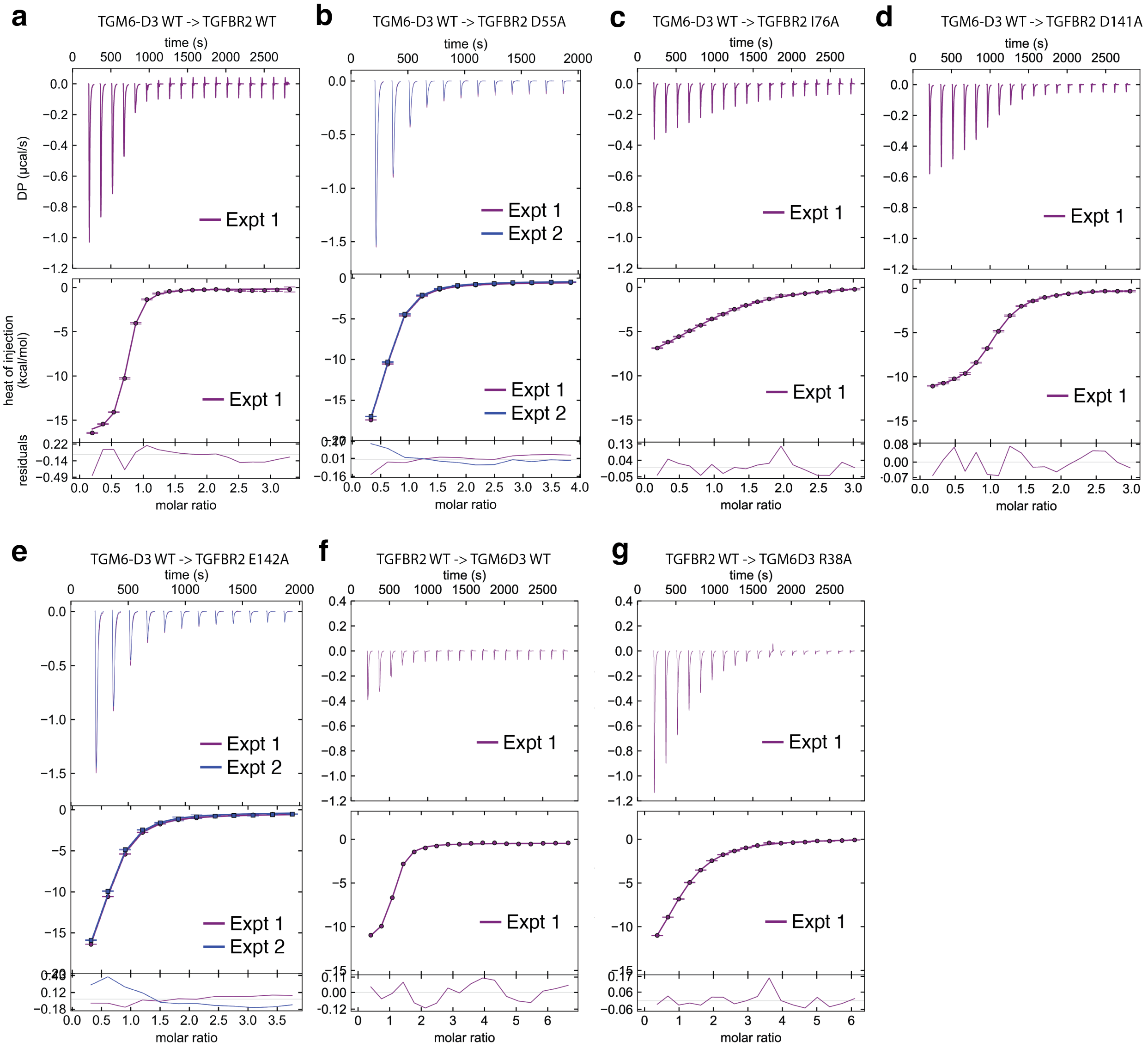

Supplementary Figure 9 continued

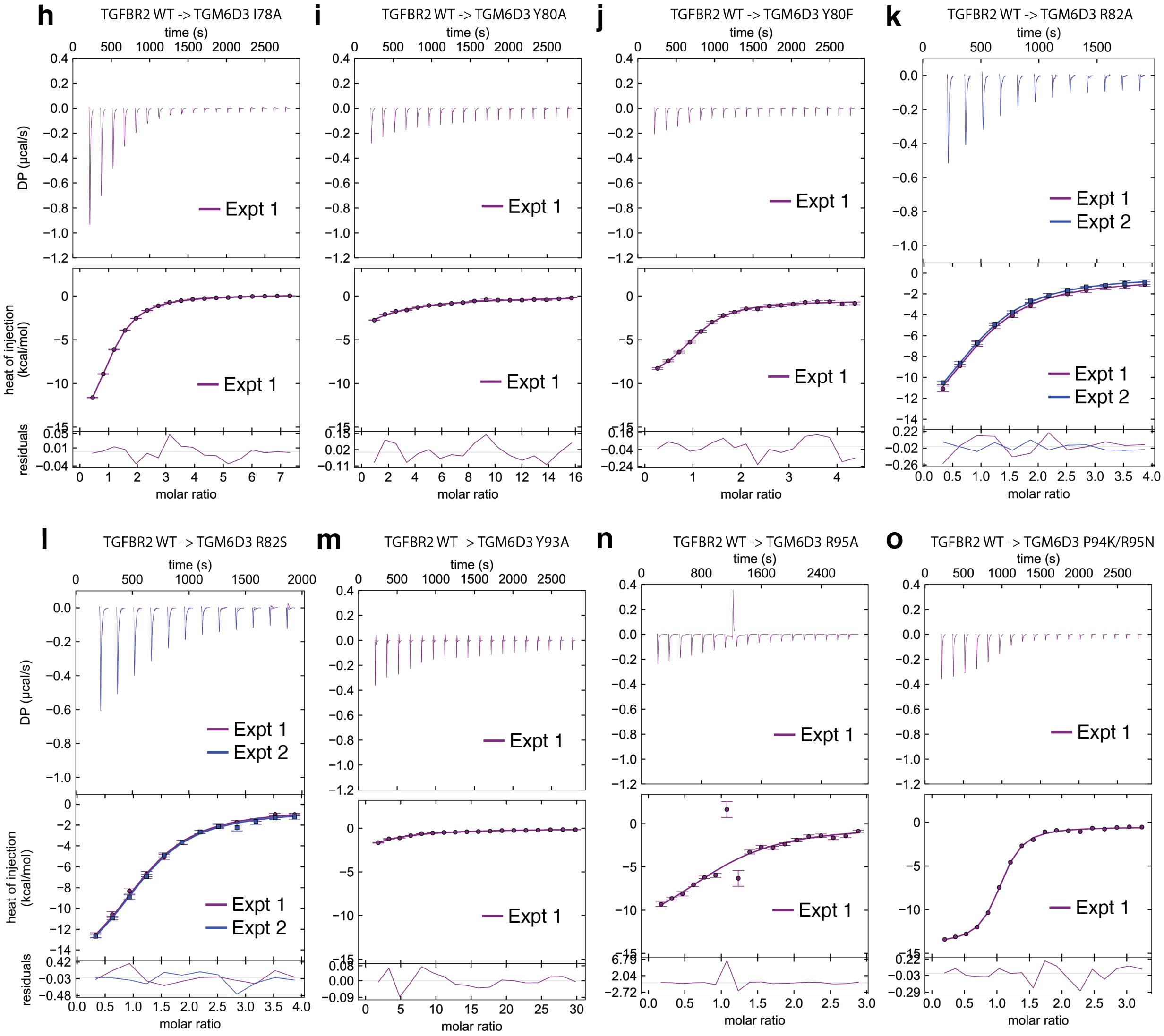

Supplementary Figure 9 continued

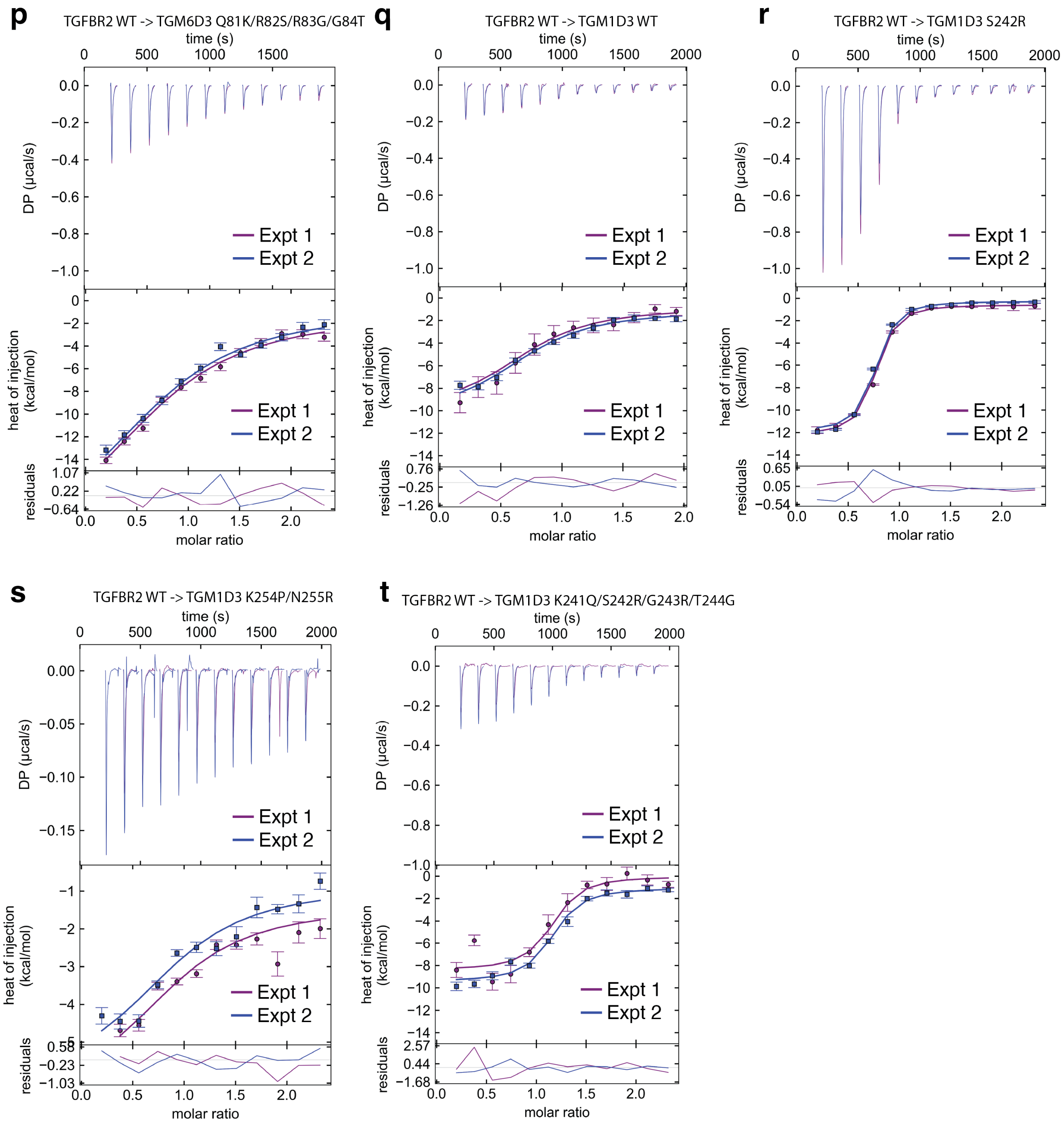

Supplementary Figure 10. 1D ^{1}H NMR spectra confirming the identity and native folding of TGM6-D3 variants. Methyl and amide regions of the 1D ^{1}H NMR spectra of the TGM6-D3 WT, I78A, Y80A, and Y93A variants used in the ITC experiments. Source data are provided through Figshare [https://doi.org/10.6084/m9.figshare.28179359].

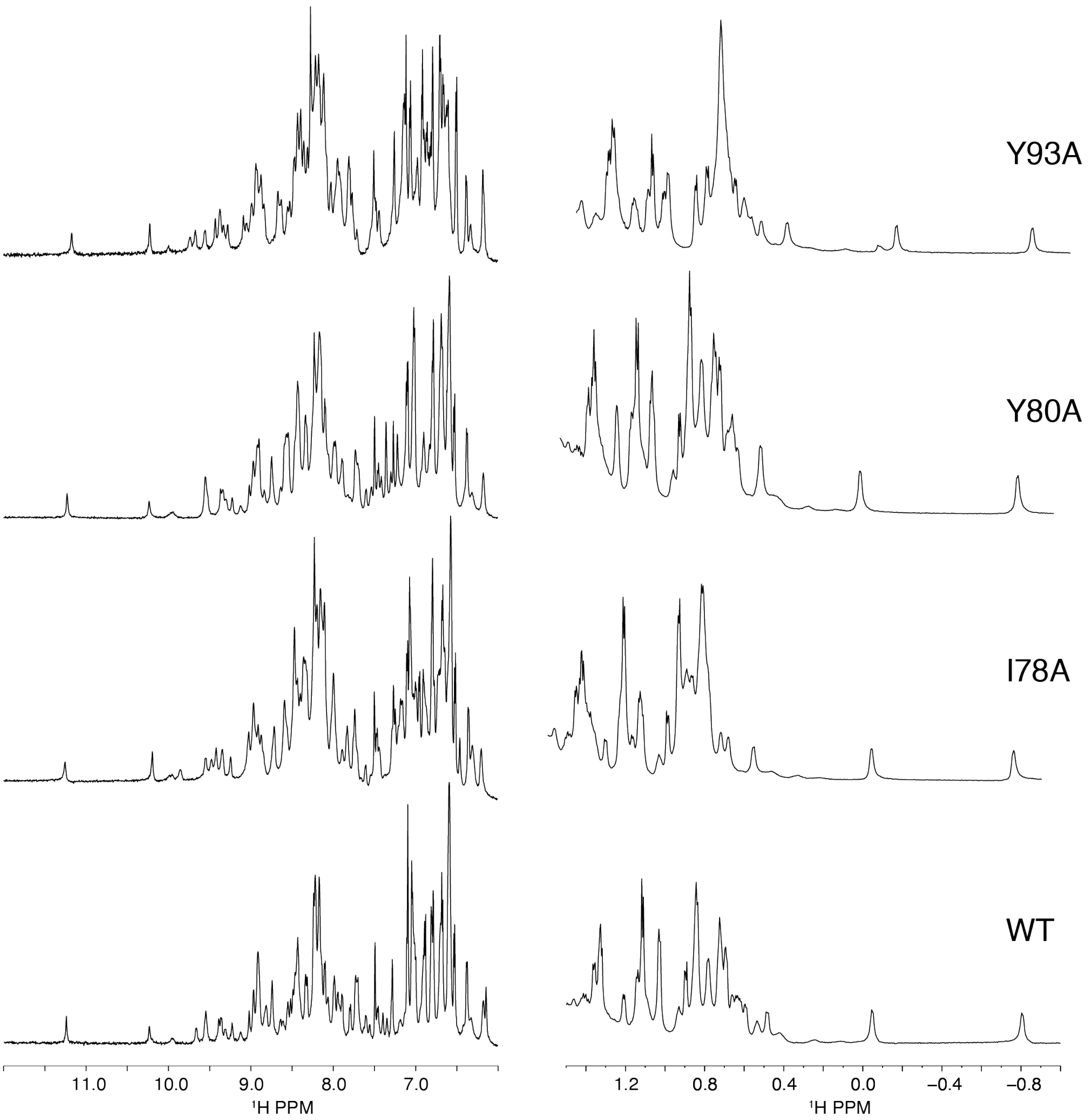

Supplementary Figure 11. Omit map of TGM6-D3 residues 82-85 with model map at different contours. (a) 0.75σ, (b) 1.00σ, (c) 1.25σ, and (d) 1.50σ. Residues that were modeled in the final structure are displayed with transparency for reference but were not included during phasing. Structural data is available through the RCSB PDB under accession code 9E9G [https://doi.org/10.2210/pdb9E9G/pdb].

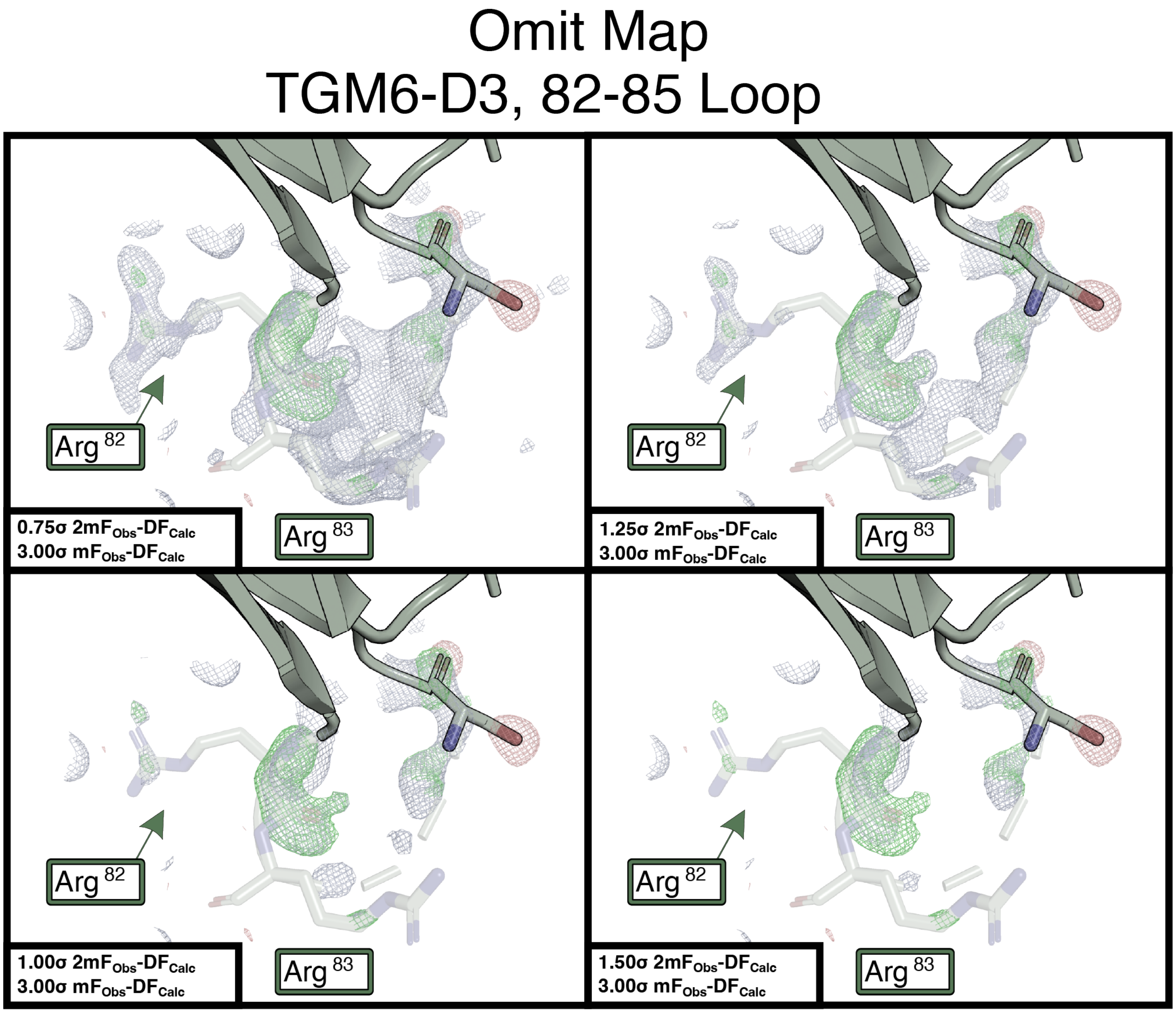

Supplementary Figure 12. Inhibition of TGM1 signaling in MFB-F11 fibroblasts. **a-c** Inhibition of TGM1 signaling in MFB-F11 fibroblasts as detected by the conversion of p-nitrophenylphosphate by secreted alkaline phosphatase (**a-b**) or by pSMAD2 Western blotting (**c**) by either TGM6 (**a,c**) or TGM6 KSGT (**b, c**). In the MFB-F11 assay, the cells were stimulated with 0, 40, or 200 pM TGM (blue, yellow, and orange symbols, respectively). Data shown in panels a and b are mean ± SD of triplicate measurements from one experiment with the same control data (0 pM TGM1). Blots shown in (**c**) include α-tubulin serving as a loading control. Source data of (**a-b**) provided as a Source Data file. Uncropped blot (**c**) provided at the end of the Supplementary Information file.

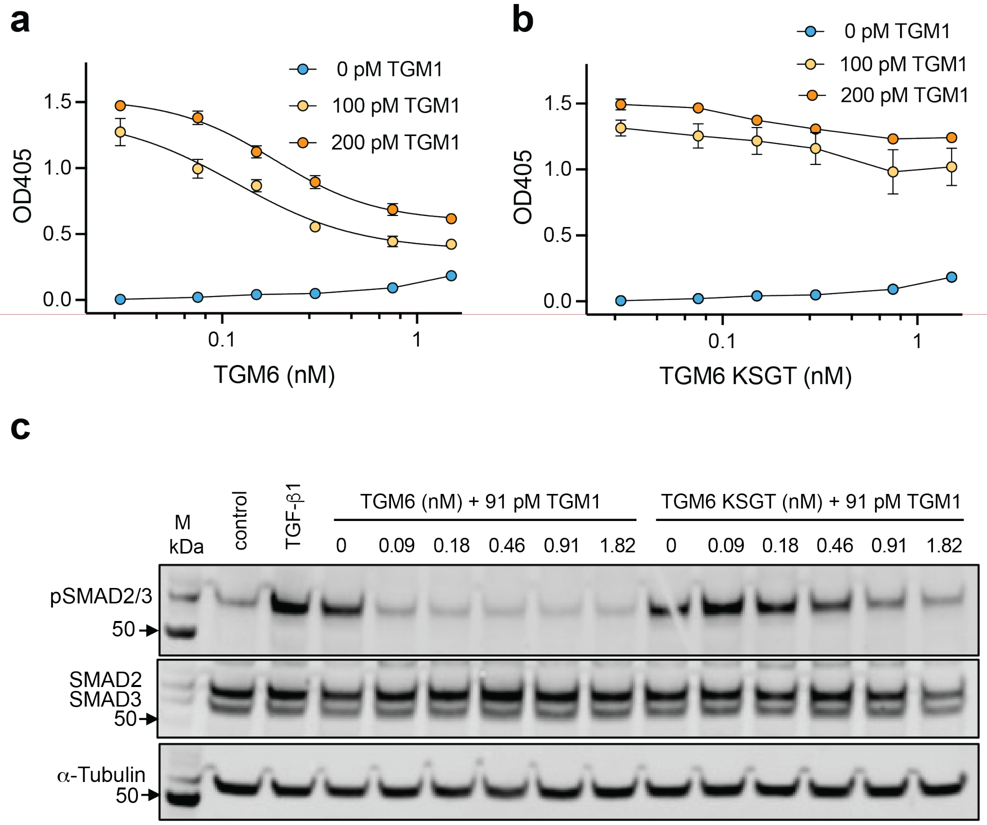

Uncropped Scan of the Western Blot shown in Supplement Figure 7a:

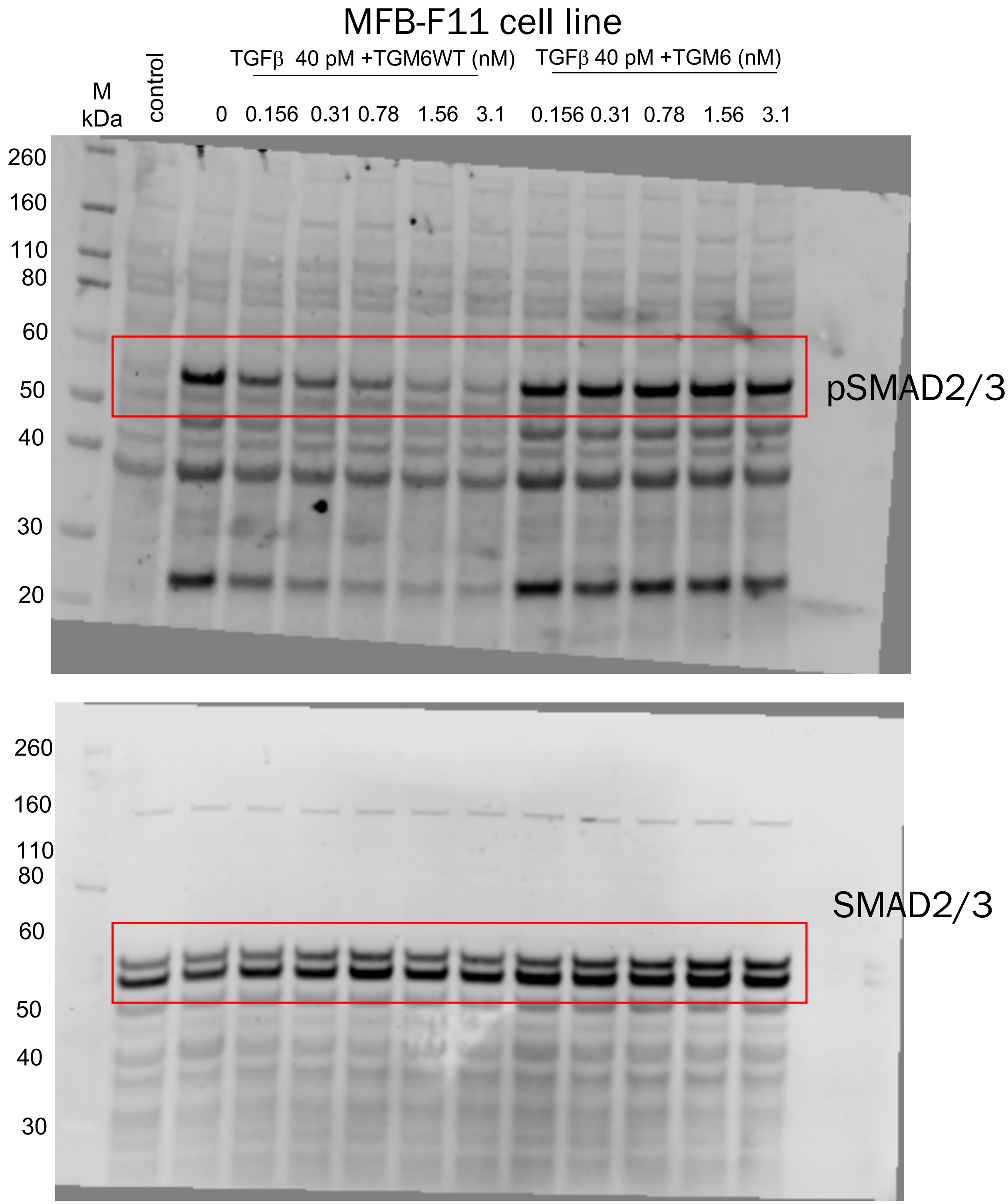

Uncropped Scan of the Western Blot shown in Supplement Figure 7b

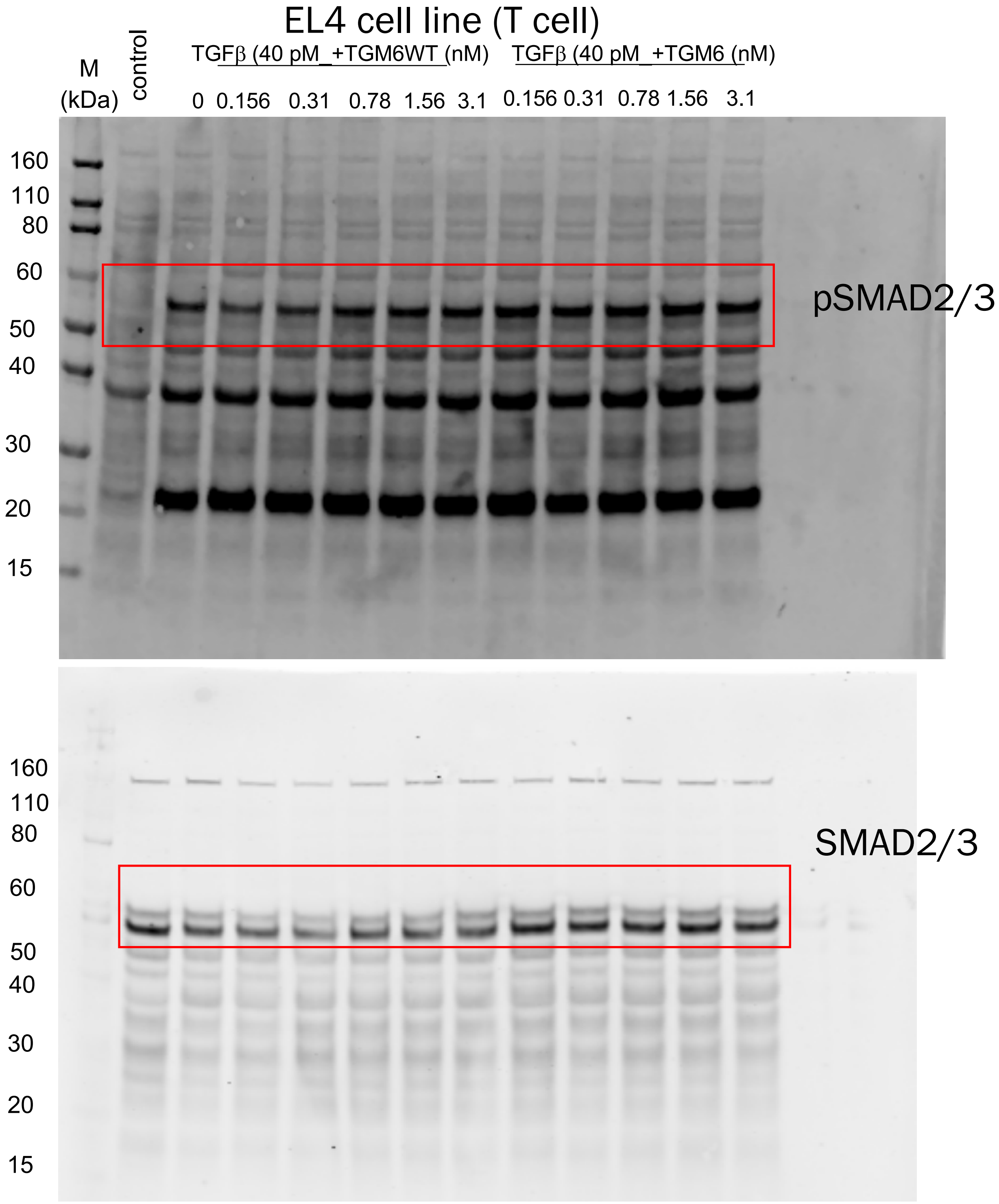

Uncropped Scan of the Western Blot shown in Supplement Figure 12c

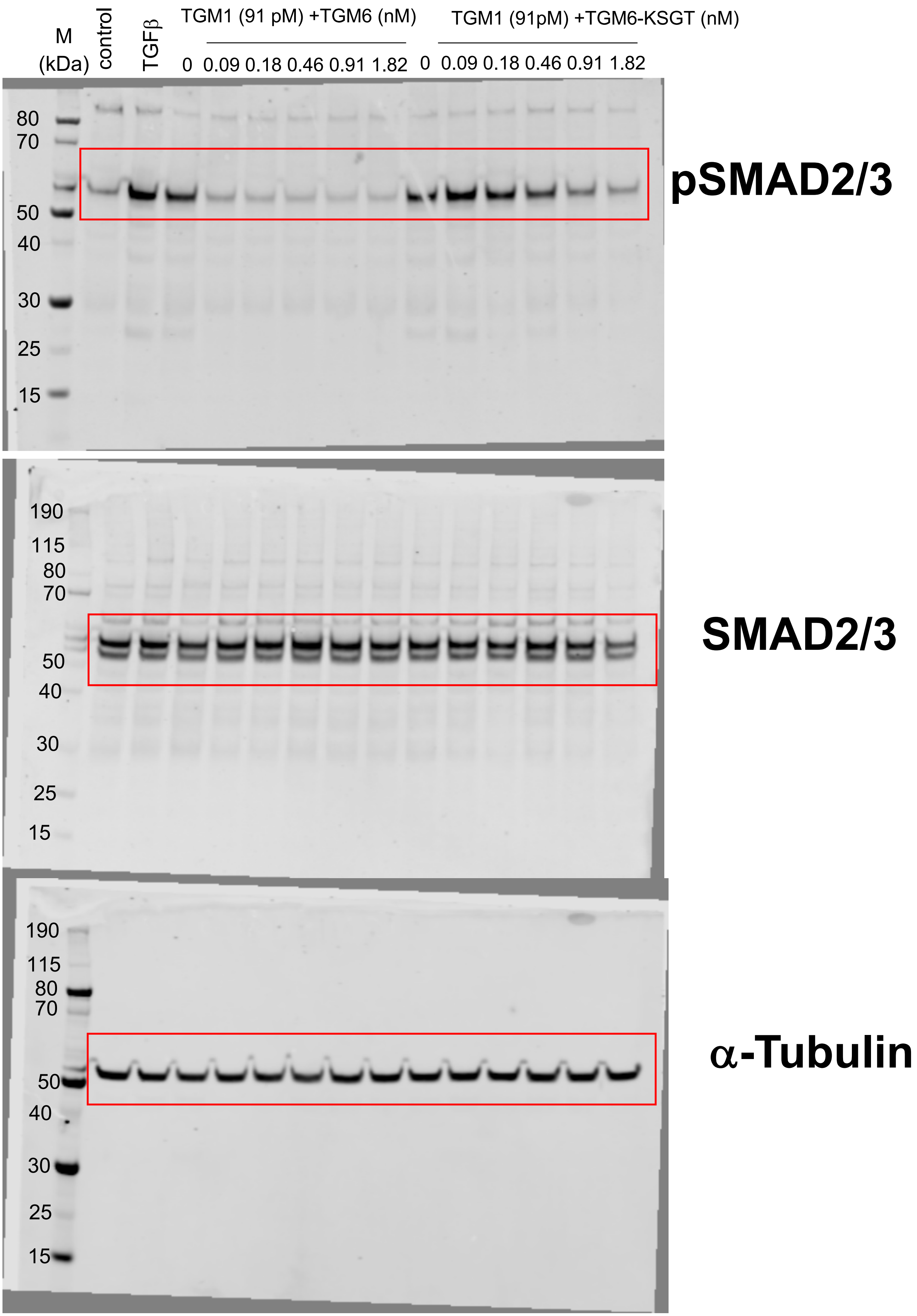

Supplement: Supplementary file 1 — Supplementary Information [file 41467_2025_56954_MOESM1_ESM.pdf]
