# Supplementary material for: TGM6 is a helminth secretory product that mimics TGF-β binding to TGFBR2 to antagonize signaling in fibroblasts
Source: Nat Commun. 2025 Feb 21;16:1847. doi: 10.1038/s41467-025-56954-z (PMC11845725; doi:10.1038/s41467-025-56954-z)
Supplement: Supplementary file 2 — Reporting Summary [file 41467_2025_56954_MOESM2_ESM.pdf]

nature portfolio

Corresponding author(s): Andrew P. Hinck

Last updated by author(s): 1/11/2025

Reporting Summary

Nature Portfolio wishes to improve the reproducibility of the work that we publish. This form provides structure for consistency and transparency in reporting. For further information on Nature Portfolio policies, see our Editorial Policies and the Editorial Policy Checklist.

Statistics

For all statistical analyses, confirm that the following items are present in the figure legend, table legend, main text, or Methods section.

n/a	Confirmed	
☐	☒	The exact sample size (*n*) for each experimental group/condition, given as a discrete number and unit of measurement
☐	☒	A statement on whether measurements were taken from distinct samples or whether the same sample was measured repeatedly
☐	☒	The statistical test(s) used AND whether they are one- or two-sided<br>*Only common tests should be described solely by name; describe more complex techniques in the Methods section.*
☒	☐	A description of all covariates tested
☒	☐	A description of any assumptions or corrections, such as tests of normality and adjustment for multiple comparisons
☐	☒	A full description of the statistical parameters including central tendency (e.g. means) or other basic estimates (e.g. regression coefficient) AND variation (e.g. standard deviation) or associated estimates of uncertainty (e.g. confidence intervals)
☐	☒	For null hypothesis testing, the test statistic (e.g. *F*, *t*, *r*) with confidence intervals, effect sizes, degrees of freedom and *P* value noted<br>*Give P values as exact values whenever suitable.*
☒	☐	For Bayesian analysis, information on the choice of priors and Markov chain Monte Carlo settings
☒	☐	For hierarchical and complex designs, identification of the appropriate level for tests and full reporting of outcomes
☒	☐	Estimates of effect sizes (e.g. Cohen's *d*, Pearson's *r*), indicating how they were calculated

Our web collection on statistics for biologists contains articles on many of the points above.

Software and code

Policy information about availability of computer code

Data collection	ITC Data: Malvern PEAQ ITC Control Software, Ver. 1.40, Malvern Instruments<br>NMR Data: Bruker Topspin, Ver 2.1, 3.1, or 3.2, Bruker Biospin<br>X-ray Data Collection: SERGUI beamline control and data collection software, Adv. Photon Source Beamline 22-ID-D, www3.ser.aps.anl.gov<br>Western blotting: LiCor biosciences image studio 5.0, LI-COR Biosciences
Data analysis	Cell Culture Data Analysis: Graphpad Prism, Ver. 10, Graphpad Inc., Referenced in M.S.<br>ITC Data: Nitpic, Sedphat, Gussi, Versions 2.1.0, 15.2b, and 1, respectively. Academic Software, Referenced in M.S.<br>NMR Data Processing: NMRPipe Ver. 9.9, Academic Software, Referenced in M.S.<br>NMR Data Analysis: NMRFAM-SPARKY Ver. 1.2_3.115, Referenced in M.S.<br>X-ray Data Analysis: Phenix.refine Ver. 1.20.1, Referenced in M.S.<br>X-ray Data Processing: XDS Ver. Jun 30, 2023, Referenced in M.S.<br>X-ray Data Analysis: Phaser Ver 2.7, Referenced in M.S.<br>X-ray Data Analysis: Coot Ver. 0.9.8.7, Referenced in M.S.<br>X-ray Data Visualization: UCSF ChimeraX Ver. 1.5, Referenced in M.S.<br>X-ray Data Visualization: Pymol Open Source Ver2.6 , Referenced in M.S.

For manuscripts utilizing custom algorithms or software that are central to the research but not yet described in published literature, software must be made available to editors and reviewers. We strongly encourage code deposition in a community repository (e.g. GitHub). See the Nature Portfolio guidelines for submitting code & software for further information.

Data

Policy information about availability of data

All manuscripts must include a data availability statement. This statement should provide the following information, where applicable:

- Accession codes, unique identifiers, or web links for publicly available datasets
- A description of any restrictions on data availability
- For clinical datasets or third party data, please ensure that the statement adheres to our policy

Source data are provided with this paper. The X-ray source data used in this study is available in the RCSB PDB database under accession code 9E9G [https://doi.org/10.2210/pdb9E9G/pdb]. The NMR and ITC source data has been deposited in the Figshare database under accession code XXX [doi.xxx]. Any additional information required to reanalyze the data reported in this paper is available from the lead contacts upon request. Plasmids generated in this study are maintained in the laboratories of Andrew Hinck (ahinck@pitt.edu), Richard M. Maizels (rick.maizels@glasgow.ac.uk), Peter ten Dijke (P.ten_Dijke@lumc.nl), and Gareth Inman (Gareth.Inman@glasgow.ac.uk) and will be made available upon request.
This paper does not report any original code.

Research involving human participants, their data, or biological material

Policy information about studies with human participants or human data. See also policy information about sex, gender (identity/presentation), and sexual orientation and race, ethnicity and racism.

Reporting on sex and gender	N/A
Reporting on race, ethnicity, or other socially relevant groupings	N/A
Population characteristics	N/A
Recruitment	N/A
Ethics oversight	N/A

Note that full information on the approval of the study protocol must also be provided in the manuscript.

Field-specific reporting

Please select the one below that is the best fit for your research. If you are not sure, read the appropriate sections before making your selection.

☒ Life sciences ☐ Behavioural & social sciences ☐ Ecological, evolutionary & environmental sciences

For a reference copy of the document with all sections, see nature.com/documents/nr-reporting-summary-flat.pdf

Life sciences study design

All studies must disclose on these points even when the disclosure is negative.

Sample size	Cell culture reporter assays: Typically triplicate measurements of the same samples; two to three independent experiments
Data exclusions	None
Replication	Cell culture reporter assays: Typically triplicate measurements of the same samples; two to three independent experiments
Randomization	Cell culture assay samples were generally assayed in order of increasing dilution
Blinding	No blinding.

Reporting for specific materials, systems and methods

We require information from authors about some types of materials, experimental systems and methods used in many studies. Here, indicate whether each material, system or method listed is relevant to your study. If you are not sure if a list item applies to your research, read the appropriate section before selecting a response.

Materials & experimental systems

n/a	Involved in the study
☐	☒ Antibodies
☐	☒ Eukaryotic cell lines
☐	☐ Palaeontology and archaeology
☐	☐ Animals and other organisms
☐	☐ Clinical data
☐	☐ Dual use research of concern
☐	☐ Plants

Methods

n/a	Involved in the study
☐	☐ ChIP-seq
☐	☐ Flow cytometry
☐	☐ MRI-based neuroimaging

Antibodies

Antibodies used

Total Smad 2/3 antibody (western blotting): SMAD2/3 Antibody #3102 from Cell signalling technologies
Phospho-Smad 2 Antibody (western blotting): Phospho-SMAD2 (Ser465/467)/SMAD3 (Ser423/425) (D27F4) Rabbit mAb#8828 from cell signalling technologies.
Alpha-Tubulin Antibody (western blotting): Anti-alpha Tubulin antibody [DM1A], Abcam, ab7291
antiMouse CD3 antibody for Treg induction assay: eBioscience Cat #14-0031-86
antiMouse TCD16/32 antibody for Treg induction assay: Biolegend, Cat # 156604
antiMouse CD4 antibody for Treg induction assay: Biolegend, Cat#BV650

Validation

Rely on Validation provided by the Manufacturer

Eukaryotic cell lines

Policy information about cell lines and Sex and Gender in Research

Cell line source(s)

NIH-3T3 CAGA-Luc reporter line: Inman Laboratory, CRUK Scotland Institute (laboratory which developed this cell line).
MFB-F11 CAGA SEAP reporter line: Wyss-Coray Laboratory, Stanford University (laboratory which developed this cell line).
NIH-3T3 CAGA GFP reporter line (Fig. 5C, S6B): ten Dijke Laboratory, Leiden University (laboratory which developed this cell line).
NIH-3T3 BRE mCHERRY reporter line (Fig. S6A): ten Dijke Laboratory, Leiden University (laboratory which developed this cell line).

Authentication

NIH-3T3 CAGA-Luc reporter line: Verification of TGF-b responsiveness.
MFB-F11 CAGA SEAP reporter line: Verification of TGF-b responsiveness.
NIH-3T3 CAGA GFP reporter line (Fig. 5C, S6B): Verification of TGF-b responsiveness.
NIH-3T3 BRE mCHERRY reporter line (Fig. S6A): Verification of BMP responsiveness.

Mycoplasma contamination

NIH-3T3 CAGA-Luc reporter line: negative (tested prior use)
MFB-F11 CAGA SEAP reporter line: Tested for mycoplasma prior to use at the institute's testing facility.
NIH-3T3 CAGA GFP reporter line (Fig. 5C, S6B): Routinely tested in the Investigators laboratory
NIH-3T3 BRE mCHERRY reporter line (Fig. S6A): Routinely tested in the Investigators laboratory

Commonly misidentified lines
(See ICLAC register)

None

Palaeontology and Archaeology

Specimen provenance

N/A

Specimen deposition

N/A

Dating methods

N/A

☐ Tick this box to confirm that the raw and calibrated dates are available in the paper or in Supplementary Information.

Ethics oversight

N/A

Note that full information on the approval of the study protocol must also be provided in the manuscript.

Animals and other research organisms

Policy information about studies involving animals; ARRIVE guidelines recommended for reporting animal research, and Sex and Gender in Research

Laboratory animals	N/A
Wild animals	N/A
Reporting on sex	N/A
Field-collected samples	N/A
Ethics oversight	N/A

Note that full information on the approval of the study protocol must also be provided in the manuscript.

Clinical data

Policy information about clinical studies
All manuscripts should comply with the ICMJE guidelines for publication of clinical research and a completed CONSORT checklist must be included with all submissions.

Clinical trial registration	N/A
Study protocol	N/A
Data collection	N/A
Outcomes	N/A

Dual use research of concern

Policy information about dual use research of concern

Hazards

Could the accidental, deliberate or reckless misuse of agents or technologies generated in the work, or the application of information presented in the manuscript, pose a threat to:

No	Yes	
☒	☐	Public health
☒	☐	National security
☒	☐	Crops and/or livestock
☒	☐	Ecosystems
☒	☐	Any other significant area

Experiments of concern

Does the work involve any of these experiments of concern:

No	Yes	
☒	☐	Demonstrate how to render a vaccine ineffective
☒	☐	Confer resistance to therapeutically useful antibiotics or antiviral agents
☒	☐	Enhance the virulence of a pathogen or render a nonpathogen virulent
☒	☐	Increase transmissibility of a pathogen
☒	☐	Alter the host range of a pathogen
☒	☐	Enable evasion of diagnostic/detection modalities
☒	☐	Enable the weaponization of a biological agent or toxin
☒	☐	Any other potentially harmful combination of experiments and agents

Plants

Seed stocks: N/A

Novel plant genotypes: N/A

Authentication: N/A

ChIP-seq

Data deposition

- [ ] Confirm that both raw and final processed data have been deposited in a public database such as GEO.
- [ ] Confirm that you have deposited or provided access to graph files (e.g. BED files) for the called peaks.

Data access links
May remain private before publication.: N/A

Files in database submission: N/A

Genome browser session
(e.g. UCSC): N/A

Methodology

Replicates: N/A

Sequencing depth: N/A

Antibodies: N/A

Peak calling parameters: N/A

Data quality: N/A

Software: N/A

Flow Cytometry

Plots

Confirm that:

- [ ] The axis labels state the marker and fluorochrome used (e.g. CD4-FITC).
- [ ] The axis scales are clearly visible. Include numbers along axes only for bottom left plot of group (a 'group' is an analysis of identical markers).
- [ ] All plots are contour plots with outliers or pseudocolor plots.
- [ ] A numerical value for number of cells or percentage (with statistics) is provided.

Methodology

Sample preparation: *Describe the sample preparation, detailing the biological source of the cells and any tissue processing steps used.*

Instrument: *Identify the instrument used for data collection, specifying make and model number.*

Software: *Describe the software used to collect and analyze the flow cytometry data. For custom code that has been deposited into a community repository, provide accession details.*

Cell population abundance: *Describe the abundance of the relevant cell populations within post-sort fractions, providing details on the purity of the samples and how it was determined.*

Gating strategy

Describe the gating strategy used for all relevant experiments, specifying the preliminary FSC/SSC gates of the starting cell population, indicating where boundaries between "positive" and "negative" staining cell populations are defined.

☐ Tick this box to confirm that a figure exemplifying the gating strategy is provided in the Supplementary Information.

Magnetic resonance imaging

Experimental design

Design type	N/A
Design specifications	N/A
Behavioral performance measures	N/A

Acquisition

Imaging type(s)	N/A
Field strength	N/A
Sequence & imaging parameters	N/A
Area of acquisition	N/A

Diffusion MRI ☐ Used ☐ Not used

Preprocessing

Preprocessing software	N/A
Normalization	N/A
Normalization template	N/A
Noise and artifact removal	N/A
Volume censoring	N/A

Statistical modeling & inference

Model type and settings	N/A
Effect(s) tested	N/A

Specify type of analysis: ☐ Whole brain ☐ ROI-based ☐ Both

Statistic type for inference (See Eklund et al. 2016)	N/A
Correction	N/A

Models & analysis

n/a	Involved in the study
☐	☐ Functional and/or effective connectivity
☐	☐ Graph analysis
☐	☐ Multivariate modeling or predictive analysis

Functional and/or effective connectivity	N/A
Graph analysis	N/A
Multivariate modeling and predictive analysis	N/A
